# Effects of Malnutrition on Perioperative Outcomes of Total Hip Arthroplasty: A Systematic Review and Meta-Analysis

**DOI:** 10.1016/j.artd.2025.101667

**Published:** 2025-04-01

**Authors:** Adam Aziz, James B. Bluhm, Tyler K. Williamson, Cameron Atkison, Andrew Eck, Chance Moore, Frank A. Buttacavoli

**Affiliations:** a University of the Incarnate Word School of Osteopathic Medicine, San Antonio, TX, USA; b Department of Orthopaedic Surgery, University of Texas Health San Antonio, San Antonio, TX, USA

## Abstract

**Background:**

Malnutrition can play a significant role in wound healing, immune response, and conditioning. The purpose of this review is to characterize definitions of malnutrition in total hip arthroplasty (THA) and analyze its effects on postoperative outcomes.

**Methods:**

A systematic search was conducted using iterations of the key terms “total hip arthroplasty” and “malnutrition.” Human studies describing malnutrition in patients undergoing primary THA for either traumatic or elective indications were included. Using the SPSS meta-analysis binary and continuous model function, the mean effect size estimate (MSE) or Cohen’s d (Cd) statistic with 95% confidence interval was reported.

**Results:**

This search yielded a total of 555 studies, of which 9 articles comprising 495,657 patients undergoing primary THA were included, characterizing 16,895 patients (3.4%) as malnourished. Studies characterized malnutrition as albumin <3.5 g/dL (n = 7) and total lymphocyte count <1500 (n = 1). Malnutrition was associated with an increased rate of nonhome discharge (MSE = 0.81, [0.55-1.07]) and likelihood of readmission (MSE = 0.86, [0.75-0.97]). Malnutrition at the time of surgery was also associated with increased rates of any complication (MSE = 1.01, [0.46-1.57]), wound complications (MSE = 1.04, [0.72-1.36]), pulmonary complications (MSE = 1.54, [1.29-1.78]), need for transfusion (MSE = 0.75, [0.54-0.96]), periprosthetic fracture (MSE = 0.65, [0.47-0.82]), reoperation (MSE = 0.72, [0.58-0.86]), and perioperative mortality (MSE = 2.05, [1.76-2.33]).

**Conclusions:**

Malnutrition was found to have significant associations with complications and disposition following THA. The findings from this meta-analysis provide support for further investigation into perioperative nutritional supplementation strategies for surgeons to optimize at-risk patients prior to THA.

**Level of Evidence:**

III.

## Introduction

Malnutrition is defined by an imbalance of vital nutrients that can result in a myriad of adverse outcomes [1]. It can affect muscle function and wound healing by inhibiting cell proliferation and metabolism, leading to the utilization of other resources within the body to provide necessary precursors via the breakdown of muscle, adipose tissue, or bone [2,3]. Furthermore, malnutrition affects the immune system through the disruption of the gut microbiome and affecting cell-building processes, which can predispose patients to infection [4]. Therefore, malnutrition is a critical modifiable risk factor that plays a major role in a perioperative setting. Nutritional adequacy has been measured in the literature by a variety of serologic markers. Several nutritional values within the literature, such as total lymphocyte count (<1500 cells/mmˆ3) and albumin level (<3.5 g/dL), have been linked to unfavorable postoperative outcomes, notably wound complications [5,6]. Understanding how this deficit may play a role in the postoperative course is important as it may serve as a modifiable aspect of management [6].

For these reasons, and as previous studies have shown, it is understood that malnutrition may have a strong association with outcomes following total hip arthroplasty (THA) [[7], [8], [9], [10], [11]]. Although THA is primarily a safe and effective procedure, postoperative complications such as dislocation (0.3%-1.4%), infection (0.3%-1.0%), periprosthetic fractures (0.1%-1.8%), and mortality (0.1%-0.4%) are becoming more common due to the increasing number of procedures. [[12], [13], [14], [15], [16], [17]]

Several studies with lower level of evidence have previously demonstrated the effect of malnutrition on outcomes of THA; however, there have been mixed reports on its effect on certain complications. In addition, few attempts have been made to investigate perioperative nutritional supplementation strategies with a strong level of evidence. Understanding the true effect of this worrisome condition is important to properly target the associated complications and focus on the outcomes where malnutrition has its greatest effects. Therefore, we sought to review the current literature to further dissect specific outcomes associated with malnutrition and THA, most notably wound complications, infection, dislocation, mortality, and reoperation. We aimed to answer 2 questions: (1) “What is the association with malnutrition and wound healing and infection-related complications following THA?” and (2) “What is the association of malnutrition with other relevant 30-day outcomes following THA?” We hypothesized that malnutrition, regardless of definition, will be positively associated with wound healing complications, infection, and risk of dislocation in patients undergoing THA.

## Material and methods

### Reporting guidelines

**Figure 1:**
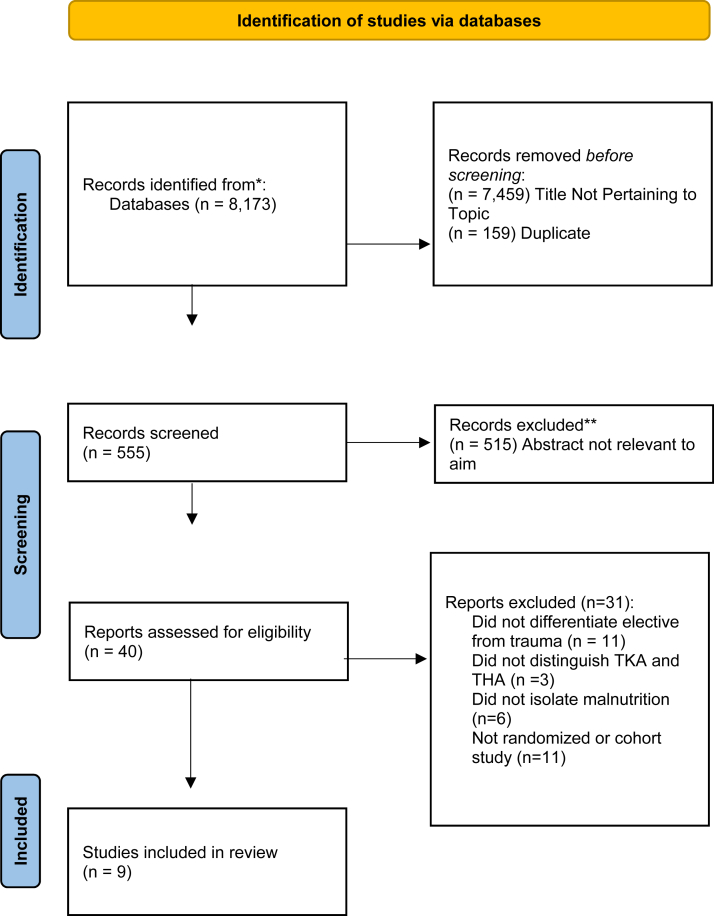
PRISMA flowchart. PRISMA, Preferred Reporting Items for Systematic Reviews and Meta-Analyses; TKA, total knee arthroplasty.

The Preferred Reporting Items for Systematic Reviews and Meta-Analyses 2020 checklist and flow diagram were used as the eligibility and inclusion criteria during the search and selection process (Fig. 1 and Supplemental Tables 1 and 2). The International Prospective Register of Systematic Reviews accepts registrations for reviews prior to conducting study searches and data extraction. The review was registered at the International Prospective Register of Systematic Reviews and is identified under CRD42024511997.

### Search and data sources

A comprehensive literature search was performed using the databases PubMed, Scopus, EmBase, and Cochrane Review with the search string of ("total hip arthroplasty" OR "total joint arthroplasty") AND ("nutrition" OR "malnutrition"). Two investigators (A.A. and J.B.B.) independently used the Rayyan system to first perform a title and abstract screen, followed by a full-text screen to identify studies that met eligibility criteria. Conflicts were resolved by a third independent investigator (T.K.W.).

### Selection criteria

The following inclusion criteria were used: (i) related to primary THA; (ii) use of either a serologic or physiological marker to assess nutritional status; (iii) reported outcomes at least 30 days postoperatively from the indicated procedure with follow-up data completed; (iv) analyzed the effect of malnutrition on outcomes related to primary hip arthroplasty procedures; (v) full text peer-reviewed; (vi) published in English language. An overview of the articles included in the study is noted in Table 1.

**Table 1:** List of articles included.

Title	Author/year	Journal	Study type	Country	Patients in study	Number of MN	Factors tested
Malnutrition as predictor of poor outcome after total hip arthroplasty	Eminovic et al., 2021 [7]	International Orthopaedics	Retrospective Cohort	Austria	220	27	Low albumin and TLC
Combined Malnutrition and Frailty Significantly Increases Complications and Mortality in Patients Undergoing Elective Total Hip Arthroplasty	Wilson et al., 2020 [18]	The Journal of Arthroplasty	Retrospective cohort	United States	105,997	3291	Low albumin
Preoperative Malnutrition and Metabolic Markers May Predict Periprosthetic Fractures in Total Arthroplasty	Lung et al., 2023 [19]	Arthroplasty Today	Retrospective Database	USA	275,107	7580	Low albumin
Preoperative malnutrition is a risk factor for delayed recovery of mobilization after total hip arthroplasty	Nanri et al., 2021 [20]	PM&R: The journal of injury, function, and rehabilitation	Retrospective Cohort	Japan	503	95	Serum albumin, total cholesterol level, and total lymphocyte count
Malnutrition increases the 30-day complication and reoperation rates in hip fracture patients treated with total hip arthroplasty	Newman et al., 2020 [21]	Hip International: The Journal of Clinical and Experimental Research on Hip Pathology and Research	Prospective	USA	1667	569	Low albumin
Effects of malnutrition on outcomes of patients with femoral head osteonecrosis undergoing total hip arthroplasty: A population-based study	Chuang et al., 2023 [22]	American Society for Parenteral and Enteral Nutrition	Retrospective Cohort	Republic of China	72,304	7152	ICD-10 code
Malnutrition Increases With Obesity and Is a Stronger Independent Risk Factor for Postoperative Complications: A Propensity-Adjusted Analysis of Total Hip Arthroplasty Patients	Fu et al., 2016 [23]	The Journal of Arthroplasty	Retrospective Cohort	USA	20,210	745	Low albumin
The Role of Malnutrition in Ninety-Day Outcomes After Total Joint Arthroplasty	Black et al., 2019 [24]	The Journal of Arthroplasty	Retrospective Cohort	USA	1989	77	Low albumin
The serum albumin threshold for increased perioperative complications after total hip arthroplasty is 3.0 g/dL	Nelson et al., 2019 [25]	Hip International	Retrospective Cohort	USA	24,586	1177	Low albumin

TLC, total lymphocyte count; MN, malnourished.

The following exclusion criteria were used: (i) not related to THA; (ii) systematic review or literature review; (iii) revision or hemiarthroplasty procedure; (iv) surgical indication was oncologic or infectious in nature; (v) studies using a measure of body mass index (BMI) as the only measure of malnutrition; (vi) does not analyze the effect of malnutrition on outcomes related to THA; (vii) not peer-reviewed; (viii) published non-English language.

### Data extraction

The following parameters, if available, were collected from each study by 2 blinded investigators: nutritional marker measure, sample size, demographics (age, sex, BMI, comorbidity status, insurance status, etc), database, surgical characteristics, and outcomes at follow-up timepoints, including length of stay, discharge disposition, complications, readmissions, reoperations, and mortalities.

### Risk of bias assessment and Grading of Recommendations Assessment, Development and Evaluation

Risk of bias was assessed via the Cochrane Risk of Bias in Nonrandomized Studies (ROBINS-I) Tool version 2.0 [26]. Using this tool, potential study biases are classified as (1) low risk of bias, (2) moderate risk of bias, or (3) serious risk of bias. The 7 potential types of biases and the method by which they are assessed include (1) bias due to confounding, (2) bias in selection of patients into the study, (3) bias in classification of interventions, (4) bias due to deviations from intended interventions, (5) bias due to missing data, (6) bias in measurement of outcomes, (7) bias in selection of the reported result and overall bias (Supplemental Table 1). Two independent assessors (A.A. and J.B.B.) performed the assessment. Disagreements were resolved through discussion or by a third reviewer (T.K.W.) if necessary.

We used the Grading of Recommendations Assessment, Development and Evaluation (GRADE) method to appraise the certainty of evidence [27,28]. Two of us (T.K.W. and A.A.) downgraded evidence based on risk of bias, inconsistency, indirectness, imprecision, and publication bias. A GRADE assessment was completed for each individual meta-analysis (Supplemental Table 2).

### Data analyses

The primary outcome was wound healing and infection-related complications following THA. The primary predictor was the form of malnutrition defined and assessed by each included study. The mean and standard deviation of values for each factor assessed were collected for all continuous variables. Meta-analysis was conducted if at least 2 studies were available for an outcome. Measures of heterogeneity used were Cochrane *Q* and the resulting χ^2^ statistic and *I*^2^. We used 95% confidence intervals to assess the amount of heterogeneity if there were at least 2 studies in the meta-analysis [29]. We incorporated all findings of the sensitivity analyses in GRADE (ie, imprecision). If meta-analysis was not possible, we used the structured reporting of effects and calculated effect sizes with a 95% confidence interval and rated the evidence according to their risk of bias [30].

Mean effect size estimates (MSEs) were calculated from final means with standard deviations and sample sizes for the malnourished and adequately nourished groups. The MSE is a standardized effect size of the malnutrition group compared to the adequately nourished group, with the MSE representing a ratio of risk related to the control. A negative value signified an advantage for the malnutrition group, whereas a positive value delineates an increased risk. Selected studies for which these or other crucial data were not directly reported or obtainable by contacting authors were not included in the review. Data were analyzed in August 2024. All analyses were performed using SPSS software (IBM Corp., IBM SPSS Statistics for Windows, v29. Armonk, NY, USA). Statistical tests were two-tailed with significance set to *P* < .05.

## Results

The searches retrieved 555 records. After screening titles and abstracts, 40 full-text assessments were carried out (Fig. 1). We included 9 studies meeting criteria (Table 1) [7,[18], [19], [20], [21], [22], [23], [24], [25]]. All studies were published in English between 2016 and 2023. The 9 clinical studies reported findings from a total of 495,657 patients undergoing THA (8 studies were able to undergo meta-analysis; 459,997 patients), of which 16,895 (3.4% overall; 3.5% in elective and 3.1% in trauma) were classified as malnourished.

Question 1: “What is the association with malnutrition and wound healing and infection-related complications following THA?”

### Wound and infectious complications

**Figure 2:**
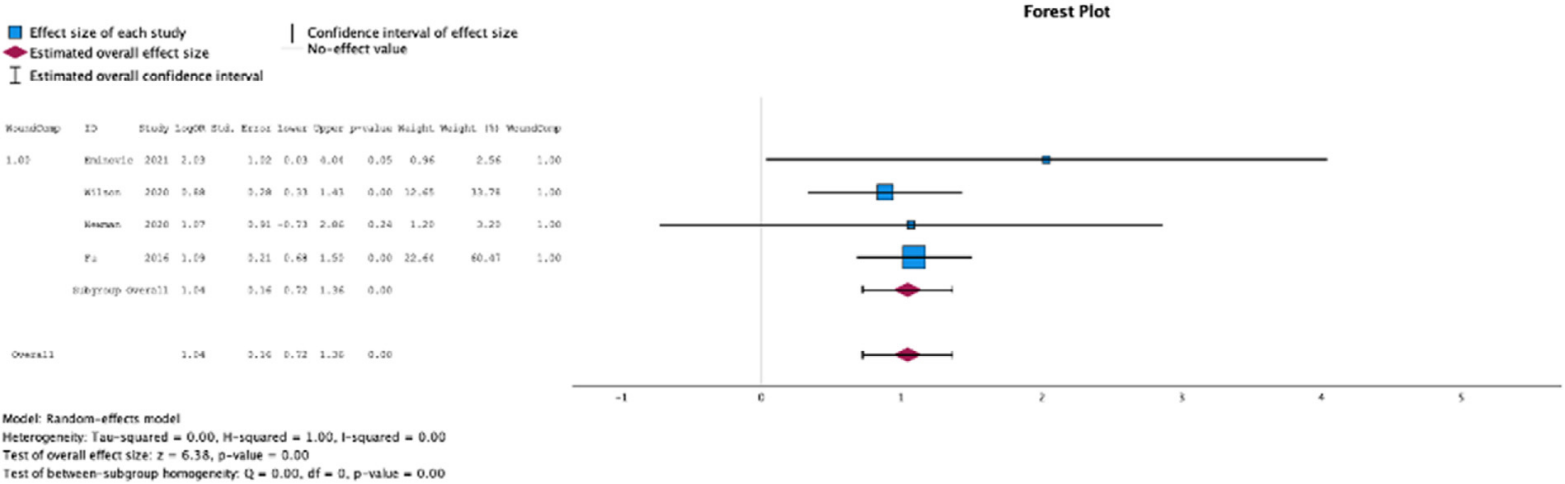
Effect of malnutrition on development of wound complications.

Four (50.0%) studies reported the relationship between malnutrition at the time of initial surgery and the development of wound complications. In these studies, malnutrition at surgical time was a risk factor for development of wound complications (MSE = 1.04, [0.72-1.36]; Z = 6.38; *P* < .001 (Fig. 2); *I*^2^ = 0%).

**Figure 3:**
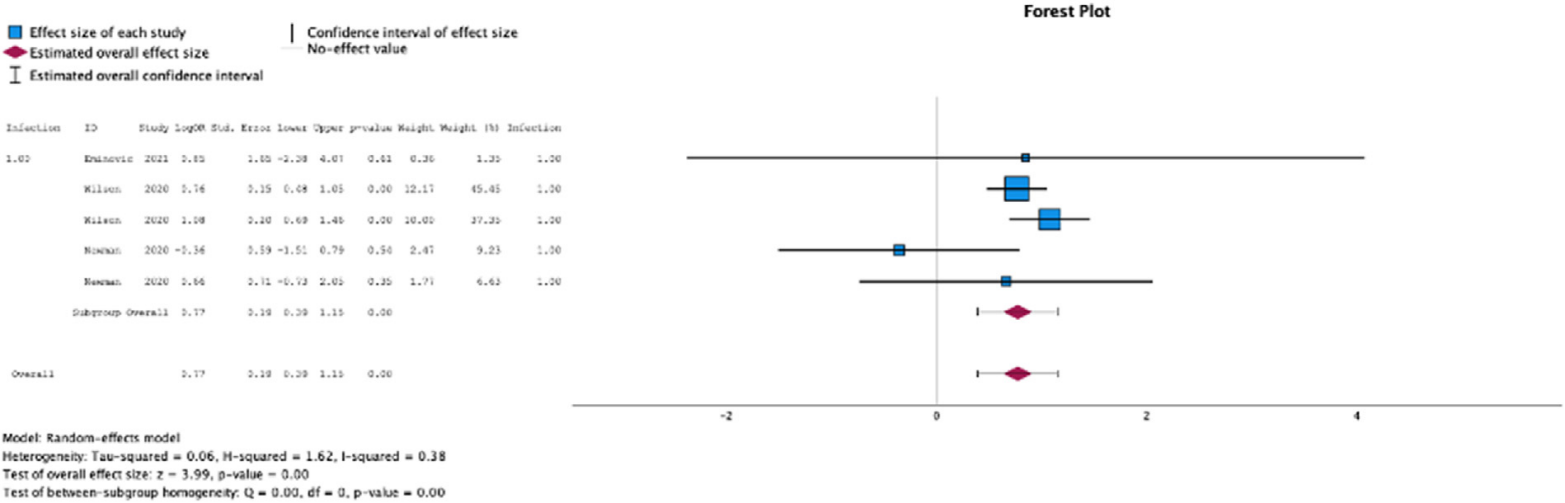
Effect of malnutrition on development of infection.

Five (62.5%) studies reported malnutrition at surgical time was a risk factor for development of either superficial or deep infections (MSE = 0.77, [0.39-1.15]; Z = 3.99; *P* < .001 (Fig. 3); *I*^2^ = 32%).

**Figure 4:**
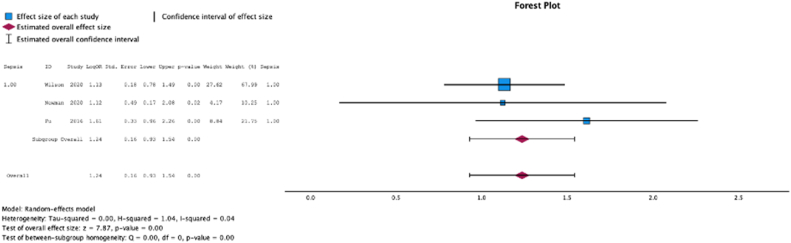
Effect of malnutrition on development of sepsis.

Three (37.5%) studies reported malnutrition at surgical time was a risk factor for development of sepsis (MSE = 1.24, [0.93-1.54]; Z = 7.87; *P* < .001 (Fig. 4); *I*^2^ = 4%).

Question 2: “What is the association of malnutrition with other relevant 30-day outcomes following THA?”

### Any complications

**Figure 5:**
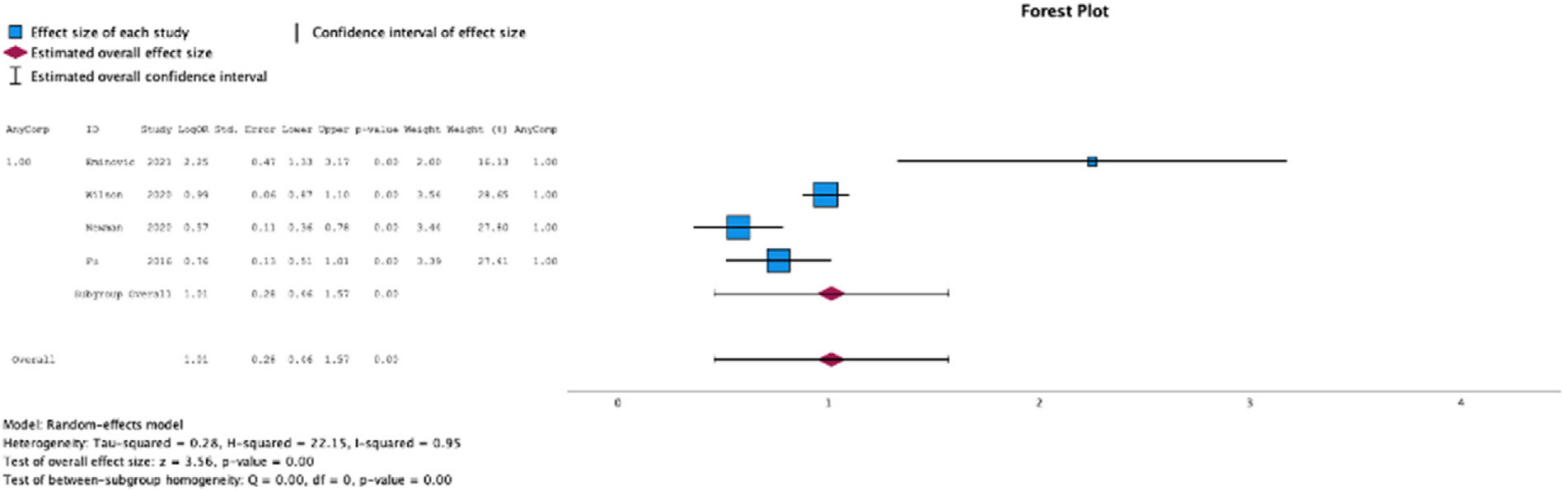
Effect of malnutrition on development of any complication.

Four (50.0%) studies reported the relationship between malnutrition at the time of initial surgery and the development of any complication. The test for heterogeneity was significant, and the studies had significant heterogeneity (*P* for heterogeneity <0.001, I2 = 95%). In these studies, malnutrition at surgical time was a risk factor for development of any complication (MSE = 1.01, [0.46-1.57]; Z = 3.56; *P* < .001 (Fig. 5)).

### Urinary complications

**Figure 6:**
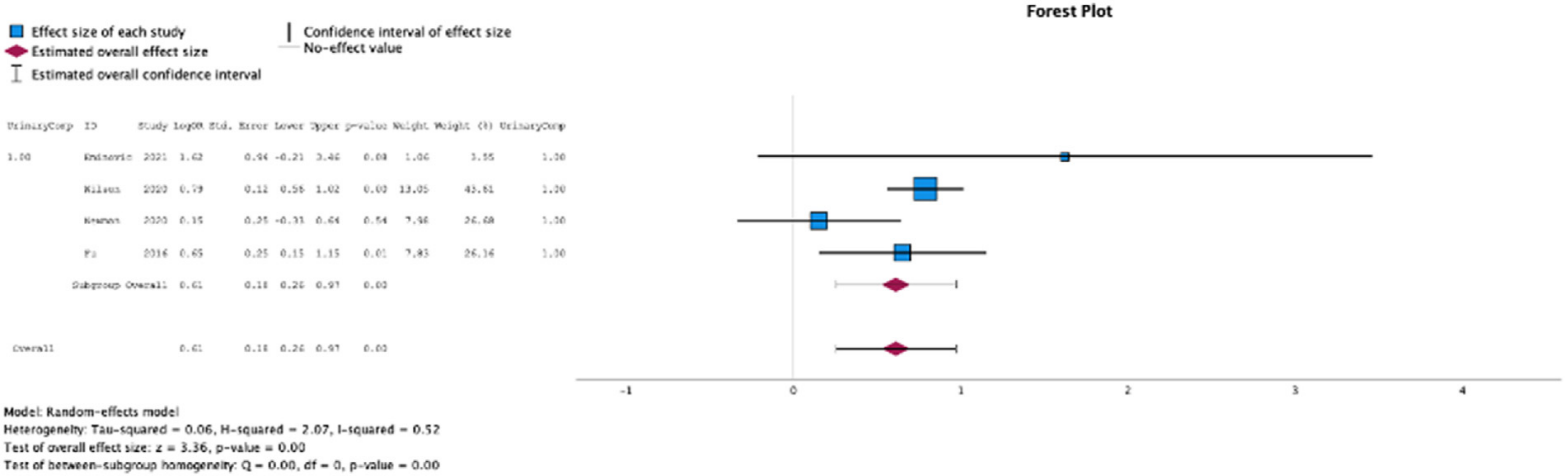
Effect of malnutrition on development of urinary complications.

Four (50.0%) studies reported the relationship between malnutrition at the time of initial surgery and the development of urinary complications. The test for heterogeneity was significant, and the studies had moderate heterogeneity (*P* for heterogeneity <0.001, I2 = 52%). In these studies, malnutrition at surgical time was a risk factor for the development of urinary complications (MSE = 0.61, [0.26-0.97]; Z = 3.36; *P* < .001 (Fig. 6)).

### Cardiopulmonary complications

**Figure 7:**
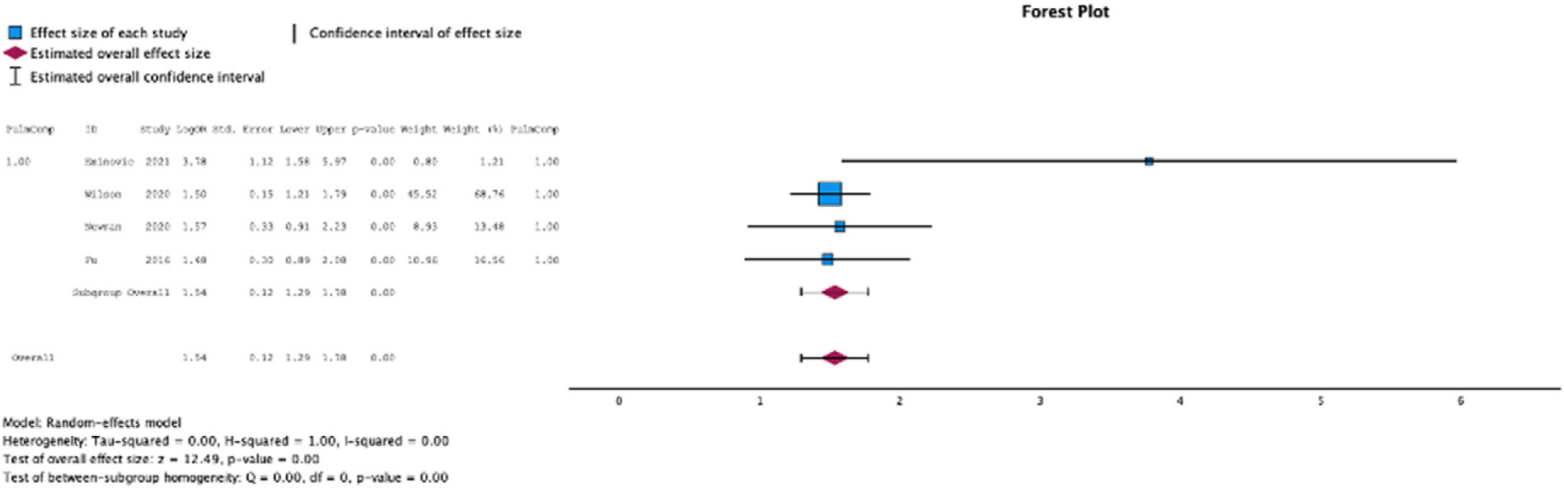
Effect of malnutrition on development of pulmonary complications.

Four (50.0%) studies reported the relationship between malnutrition at the time of initial surgery and the development of pulmonary complications. In these studies, malnutrition at surgical time was a risk factor for the development of pulmonary complications (MSE = 1.54, [1.29-1.78]; Z = 12.49; *P* < .001 (Fig. 7); *I*^2^ = 0%).

**Figure 8:**
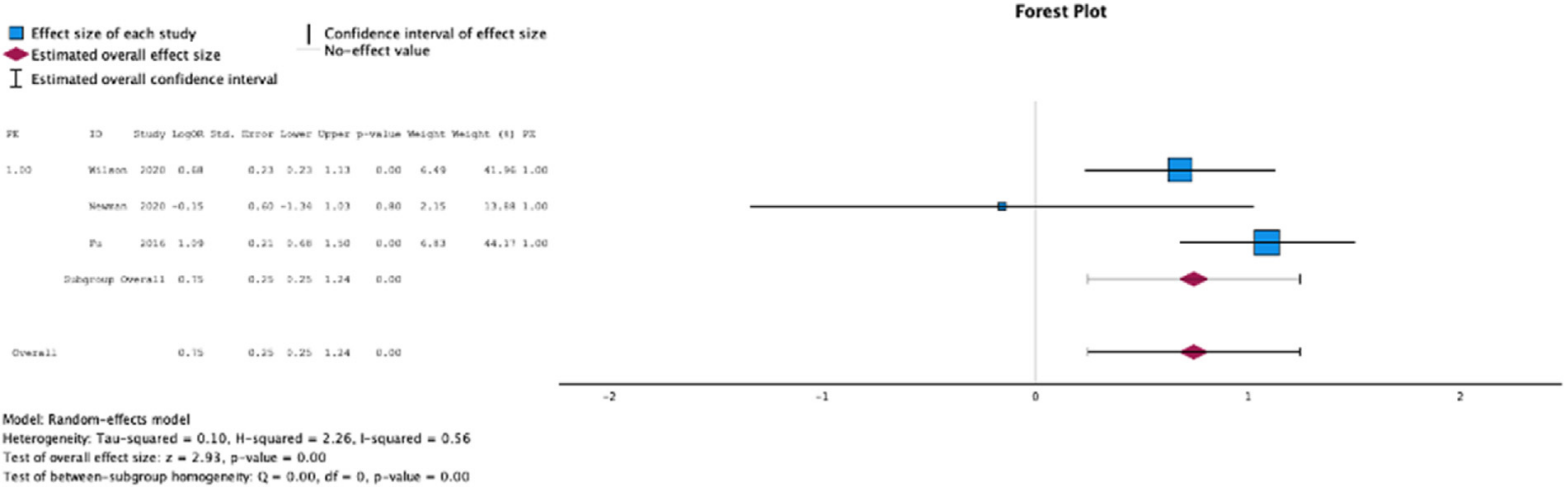
Effect of malnutrition on development of pulmonary embolism.

Three (37.5%) studies reported malnutrition at surgical time was a risk factor for the development of pulmonary embolism (MSE = 0.75, [0.25-1.24]; Z = 2.93; *P* < .001 (Fig. 8); *I*^2^ = 56%).

**Figure 9:**
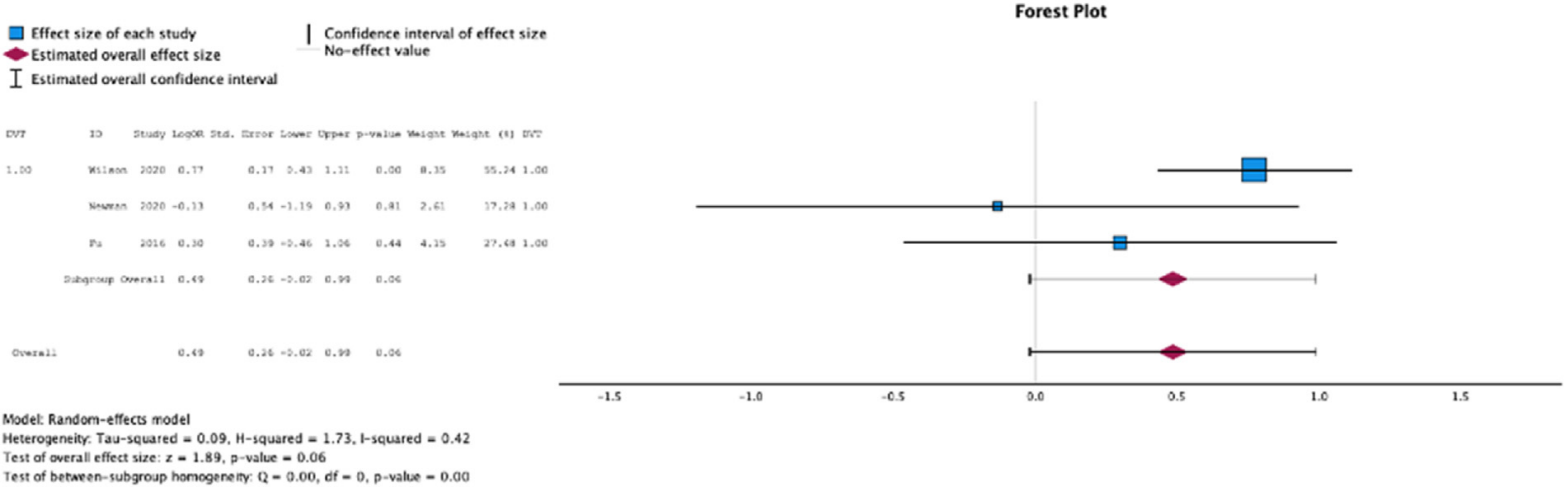
Effect of malnutrition on development of deep vein thrombosis.

Three (37.5%) studies reported malnutrition at surgical time was not a risk factor for the development of deep vein thrombosis (MSE = 0.49, [(−0.02) to 0.99]; Z = 1.89; *P* = .06 (Fig. 9); *I*^2^ = 42%).

**Figure 10:**
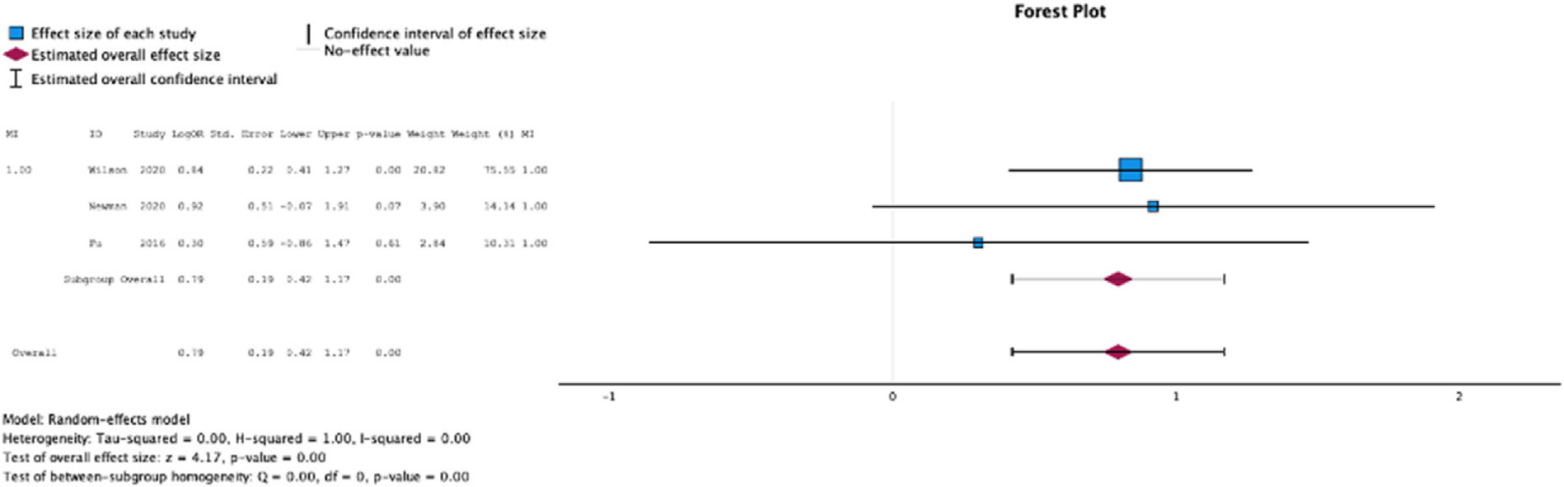
Effect of malnutrition on development of myocardial infarction.

Three (37.5%) studies reported malnutrition at surgical time was a risk factor for the development of myocardial infarction (MSE = 0.79, [0.42-1.17]; Z = 4.17; *P* < .001 (Fig. 10); *I*^2^ = 0%).

**Figure 11:**
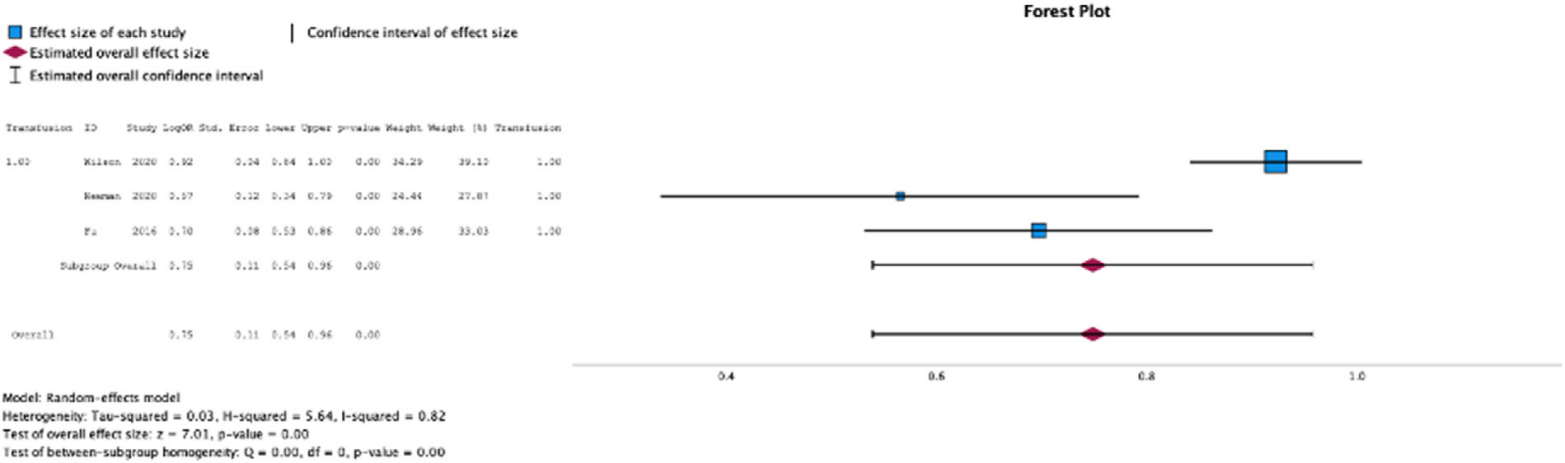
Effect of malnutrition on need for postoperative transfusion.

Three (37.5%) studies reported malnutrition at surgical time was a risk factor for requiring a postoperative blood transfusion (MSE = 0.75, [0.54-0.96]; Z = 7.01; *P* < .001 (Fig. 11)*; I*^2^ = 82%).

### Disposition and readmission

**Figure 12:**
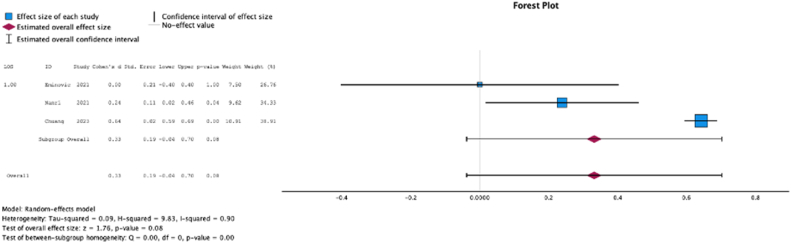
Effect of malnutrition on hospital length of stay.

Three (37.5%) studies reported the relationship between malnutrition at the time of initial surgery and hospital length of stay. In these studies, malnutrition at surgical time was not associated with increased length of stay (Cohen’s d = 0.33, [{−0.04} to 0.70]; Z = 1.76; *P* = .08 (Fig. 12)); *I*^2^ = 90%).

**Figure 13:**
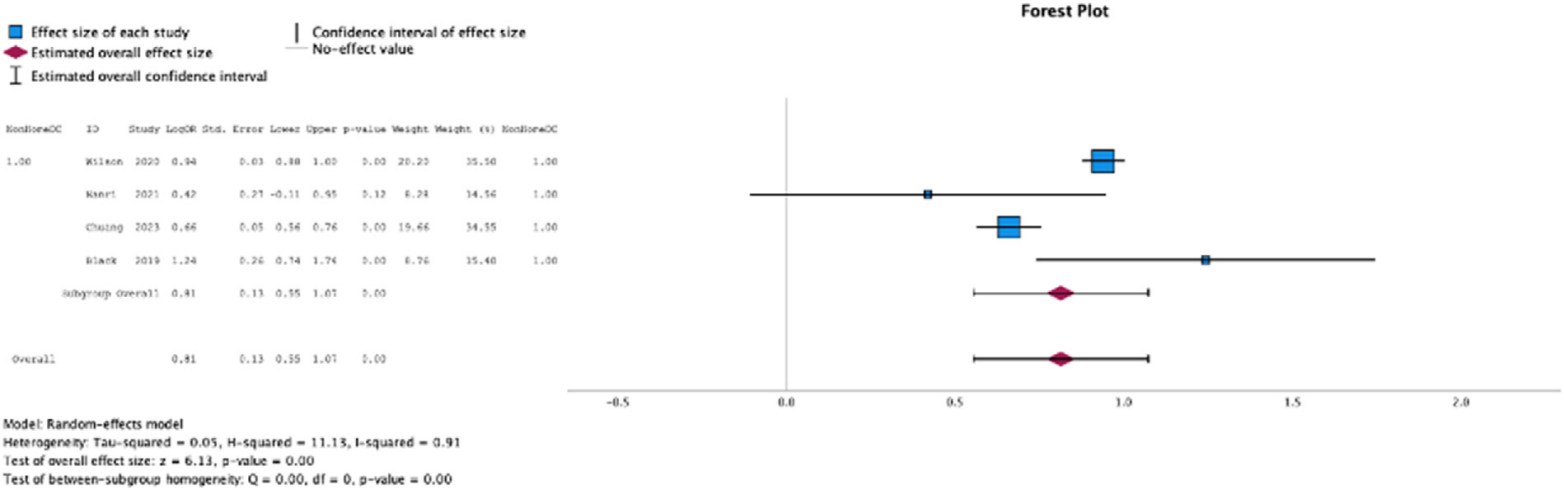
Effect of malnutrition on nonhome discharge.

Four (50.0%) studies reported malnutrition at surgical time was a risk factor for nonhome discharge (MSE = 0.81, [0.55-1.07]; Z = 6.13; *P* < .001 (Fig. 13); *I*^2^ = 52%).

**Figure 14:**
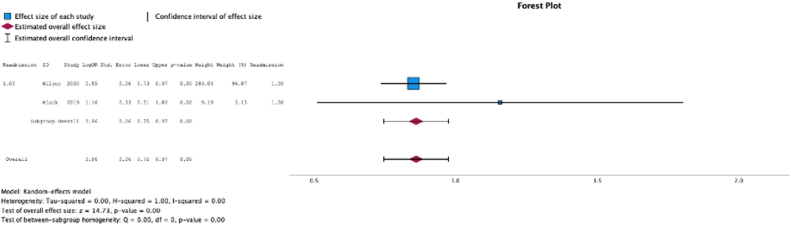
Effect of malnutrition on readmission.

Two (25.0%) studies reported malnutrition at surgical time was a risk factor for readmission (MSE = 0.86, [0.75-0.97]; Z = 14.73; *P* < .001 (Fig. 14); *I*^2^ = 0%).

### Periprosthetic fracture, reoperation, and mortality

**Figure 15:**
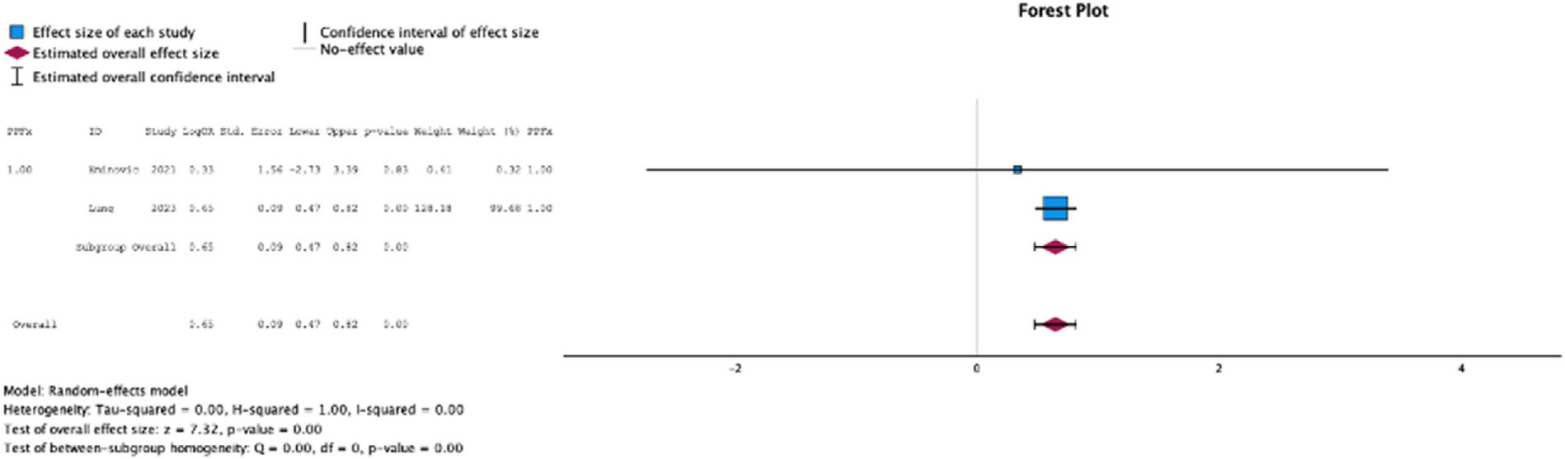
Effect of malnutrition on development of periprosthetic fracture.

Two (25.0%) studies reported the relationship between malnutrition at the time of initial surgery and the development of periprosthetic fracture. In these studies, malnutrition at surgical time was a risk factor for periprosthetic fracture (MSE = 0.65, [0.47-0.82]; Z = 7.32; *P* < .001 (Fig. 15); *I*^2^ = 0%).

**Figure 16:**
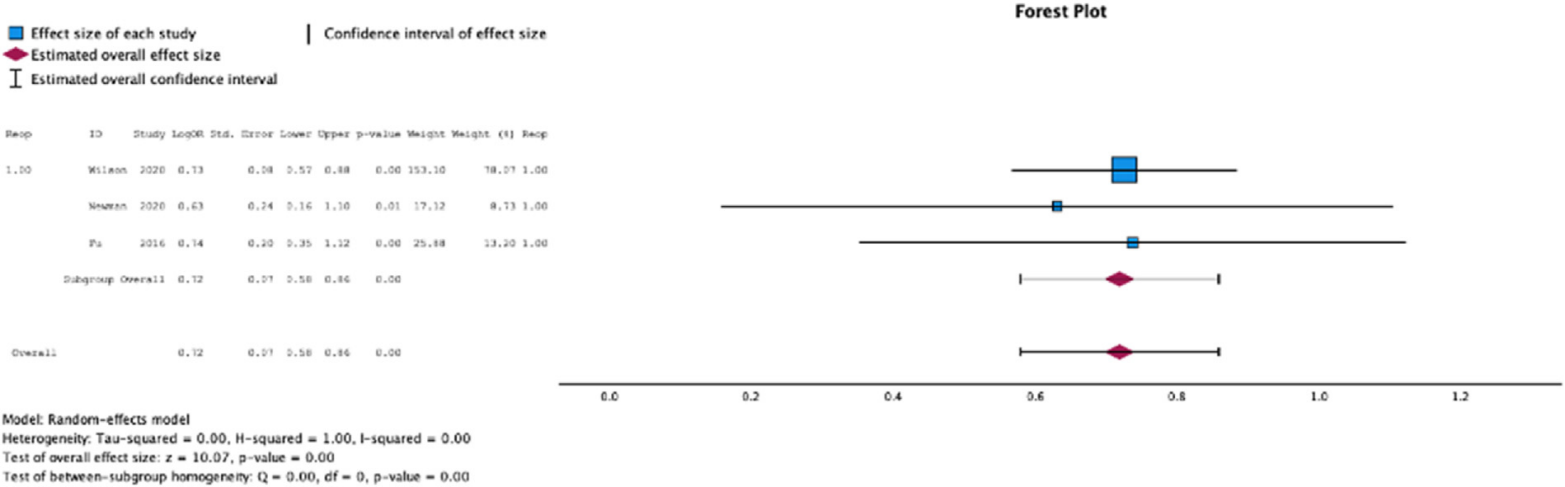
Effect of malnutrition on readmission.

Three (37.5%) studies reported malnutrition at surgical time was a risk factor for reoperation (MSE = 0.72, [0.58-0.86]; Z = 10.07; *P* < .001 (Fig. 16); *I*^2^ = 0%).

**Figure 17:**
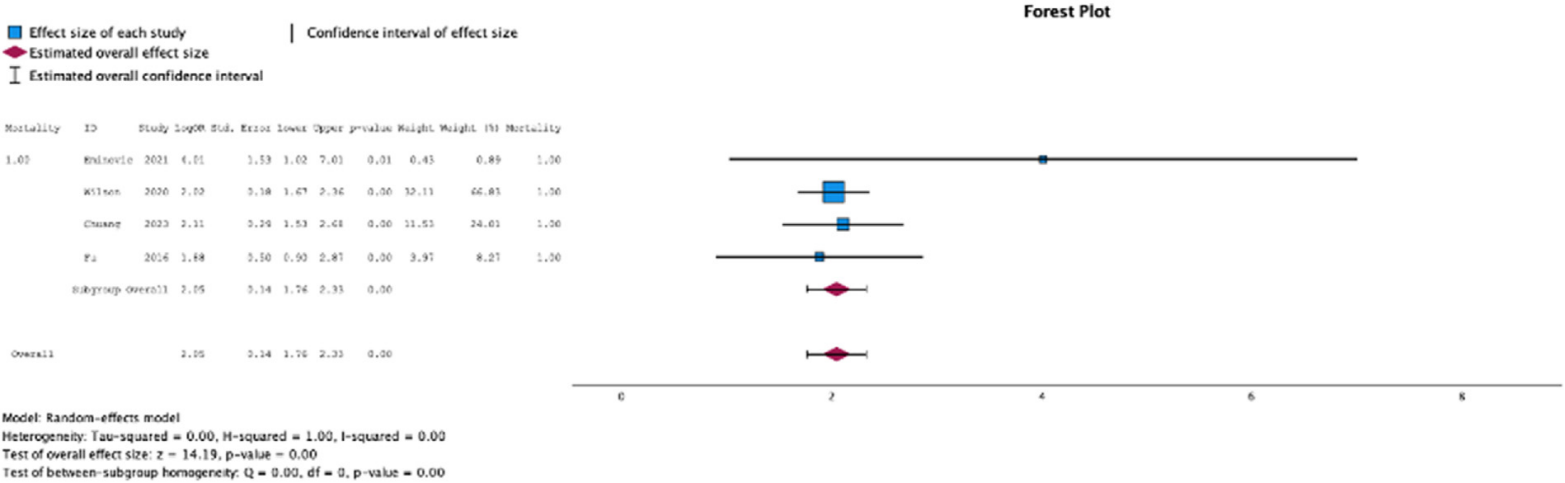
Effect of malnutrition on mortality.

Four (50.0%) studies reported malnutrition at surgical time was a risk factor for mortality (MSE = 2.05, [1.76-2.33]; Z = 14.19; *P* < .001 (Fig. 17); *I*^2^ = 0%).

## Discussion

Malnutrition has been shown to be associated with poor outcomes in various surgical settings, leading to impaired wound healing and immune dysregulation and placing patients at risk for postoperative complications [31]. Although this association is well known, the definitions of malnutrition have not been well defined, specifically in THA literature. While certain studies use markers like albumin and lymphocyte count, others rely on BMI and other indices or diagnosis codes [32]. The goal of our study was to highlight the current definitions for malnutrition and analyze their associations with postoperative outcomes following THA. Our systematic review revealed 9 eligible studies demonstrating preoperative malnutrition may be associated with increased rates of problematic complications, such as infection, impaired wound healing, and periprosthetic fracture, as well as both reoperation and mortality following THA.

Our results showed that 3.4% of patients undergoing primary THA were considered to be malnourished, which is lower than the 12.3% noted in previous studies [33,34]. These patients were found to have an association with complications affecting critical systems, such as pulmonary embolism and myocardial infarction. Previous studies have correlated these findings with the respiratory muscle weakness and compromised respiratory function observed in malnourished individuals, increasing their risk for pneumonia and other pulmonary-related complications [35,36]. In addition, other studies have noted increased rates of mortality in malnourished patients due to acute myocardial infarctions [36]. Furthermore, previous studies have also identified the role of malnutrition in urinary complications in patients with hip fractures and may play a similar role in this population [37]. While most understand infection and impaired wound healing are primarily involved in the setting of malnutrition, these complications underscore malnutrition that may have indirect systemic effects following THA.

While those complications are often more related to mortality, other complications can serve as the precursor for revision surgery. Aseptic loosening and osteolysis remain a primary concern within the postoperative follow-up [38]. Given the importance of ingrowth and ongrowth onto the implant for stability, nutritional status can directly affect the bone-implant interface, as well as stability due to the decreased soft tissue mass and interval healing [[39], [40], [41], [42], [43]]. While we were unable to directly observe these types of complications within our meta-analysis, patients with malnutrition were at increased risk for reoperation overall and, therefore, may be at additional risk for these precluding events. Conversely, we were able to demonstrate increased likelihood of periprosthetic fracture following THA for malnourished patients, as evidenced in previous studies [43,44]. As malnutrition and bone quality are directly related, it is no surprise these patients are at risk for bony complications following THA [45]. Understanding these findings allows for better communication to the patient of the risks associated with THA and further justification to the patient of the importance of nutritional adequacy prior to proceeding with surgical intervention.

Lymphocytes play a strong role in inflammatory processes to help regulate cytokines, mediate inflammation throughout the body, and are directly affected by inadequate nutrition [46]. When lymphocyte count is decreased and the ability to mount an immune response is limited, Tsantes et al demonstrate malnutrition plays a crucial role in increased infection rates in patients who underwent THA [47]. Furthermore, septic revision has a high association with mortality, and recent studies have highlighted that those with certain comorbidities including malnutrition undergoing septic revision are most at risk [48]. Therefore, preventative methods should be taken preoperatively and intraoperatively to guard directly against infection and optimize physiologic states like nutritional status to minimize risk of this precursor to postoperative mortality.

In summary, these findings reveal that malnutrition is a significant risk factor for a wide range of postoperative complications in THA patients, including mortality. Given the consistency of its impact across studies and complication types, preoperative nutritional optimization should be prioritized in this surgical population. Previous studies have noted the effect of intervention in the perioperative period after THA, with significant improvement in postoperative markers, complication prevention, and cost-effectiveness of the procedure seen in randomized controlled trials and prospective cohort studies [[49], [50], [51], [52], [53]]. In previous literature, it mainly consisted of carbohydrate repletion; however, certain studies indicated the patients who most often prevented complications were those that responded with increased protein content. Other subspecialties within orthopaedics, such as spine surgery, have also made strides to investigate nutritional intervention and have found similar effects, especially in wound and infection-related complications [6]. Therefore, orthopaedic surgeons should focus on improving their nutritional status either directly by providing supplementation or directing the patient to a comprehensive nutritional program with historical adherence. Although THA has been shown to produce good outcomes in upward of 95% of patients, the sheer rate of procedures presents a large number of patients who may sustain complications, and providing adequate nutrition may greatly impact those experiencing postoperative complications.

### Limitations

Significant heterogeneity in certain outcomes was seen and may limit the generalizability and direct associations with their relationship to malnutrition due to other confounding effects. Our review is limited by the studies currently available, and additional or more detailed nutritional markers (prealbumin, vitamin D, transferrin, fatty acids, etc) either were not directly assessed or did not meet criteria for inclusion within our study [53]. The outcomes investigated within our review may differ in definition across studies included and may further contribute to the heterogeneity. Use of retrospective data for the majority of these studies may also affect the strength of our findings and increase bias. Different preoperative variables were recorded across all studies and limited our ability to perform a meta-regression to control for other mitigating factors related to outcomes. In addition, future studies could explore specific nutritional interventions and optimal thresholds for hypoalbuminemia and total lymphocyte count, as well as other markers of malnutrition to mitigate these risks, and seek preoperative strategies to mitigate malnutrition prior to surgery to improve postoperative recovery and reduce complication rates in patients undergoing THA.

## Conclusions

This analysis reinforces the critical importance of addressing malnutrition in patients undergoing THA. The associations between malnutrition and a range of complications, from wound infections to mortality, emphasize the need for preoperative nutritional screening and the presumed utility of perioperative nutritional intervention. The findings suggest that improving nutritional status could significantly reduce postoperative risk, improve overall recovery in this at-risk patient population, and further enhance the efficacy of THA outcomes overall.

## Funding

Funding (APC fee) was provided by the Department of Orthopaedic Surgery at the University of Texas Health San Antonio.

## Conflicts of interest

C. Moore is a paid consultant for Zimmer. F. A. Buttacavoli is a paid consultant for Heraeus, KCI, Medtronic, Sanara MedTech, and Zimmer. T. K. Williamson is a member of the AAOS Resident Assembly Research Committee and is an editorial board member for the Journal of Orthopaedic Experience and Innovation. All other authors declare no potential conflicts of interest.

For full disclosure statements refer to https://doi.org/10.1016/j.artd.2025.101667.

## CRediT authorship contribution statement

**Adam Aziz:** Writing – original draft, Validation, Methodology, Investigation, Conceptualization. **James B. Bluhm:** Writing – original draft, Validation, Methodology, Investigation, Conceptualization. **Tyler K. Williamson:** Writing – original draft, Resources, Methodology, Investigation, Formal analysis, Data curation, Conceptualization. **Cameron Atkison:** Writing – review & editing, Validation, Methodology, Investigation. **Andrew Eck:** Writing – review & editing, Validation, Methodology, Investigation. **Chance Moore:** Writing – review & editing, Visualization, Investigation, Conceptualization. **Frank A. Buttacavoli:** Writing – review & editing, Validation, Supervision, Project administration, Methodology, Investigation, Conceptualization.

## Figures and Tables

**Supplemental Table 1:** Risk of bias (ROBINS-I) assessment of included articles.

Study	Confounding	Patient selection	Classification	Deviation	Missing data	Outcome measures	Result selection	Overall
Eminovic et al. [7]	M	M	L	L	M	L	M	M
Wilson et al. [18]	M	L	L	L	M	L	L	L
Lung et al. [19]	M	L	L	L	M	L	L	L
Nanri et al. [20]	M	M	L	L	M	M	M	M
Newman et al. [21]	M	M	L	L	M	M	M	M
Chuang et al. [22]	M	L	L	L	M	M	L	L
Fu et al. [23]	M	M	L	L	M	M	M	M
Black et al. [24]	M	L	L	L	M	L	L	L
Nelson et al. [25]	M	L	L	L	M	L	L	L

L, low risk; M, medium risk.

**Supplemental Table 2:** GRADE assessment of included articles.

Malnutrition definition	Number of patients (studies)	Quality of the evidence (GRADE)
Albumin (<3.5 g/dL)	15,143 (Mariconda et al.)	Moderate ⊕⊕⊕◯ (due to risk of bias)
Total lymphocyte count	27 (Saunders and Smith)	Moderate ⊕⊕⊕◯ (due to risk of bias)
ICD-10 codes	1752 (Saunders and Smith)	Moderate ⊕⊕⊕◯ (due to risk of bias)

## Appendix A Supplementary data

Conflict of Interest Statement for Aziz

Conflict of Interest Statement for Eck

Conflict of Interest Statement for Atkison

Conflict of Interest Statement for Moore

Conflict of Interest Statement for Buttacavoli

Conflict of Interest Statement for Bluhm

Conflict of Interest Statement for Williamson
