# Supplementary material for: Effects of Malnutrition on Perioperative Outcomes of Total Hip Arthroplasty: A Systematic Review and Meta-Analysis
Source: Arthroplast Today. 2025 Apr 1;33:101667. doi: 10.1016/j.artd.2025.101667 (PMC11999226; doi:10.1016/j.artd.2025.101667)
Supplement: Conflict of Interest Statement for Atkison [file mmc3.docx]

# INDIVIDUAL CONFLICT OF INTEREST STATEMENT

***American Association of Hip and Knee Surgeons***

(Adopted from the American Academy of Orthopaedic Surgeons disclosure statement)

The following form **must be filled out completely and submitted by each author (example, 6 authors, 6 forms).**

**All items require a response. If there is no relevant disclosure for a given item, enter "*None*.”**

**Manuscript Title:** Effects of Malnutrition on Perioperative Outcomes of Total Hip Arthroplasty: A Systematic Review and Meta-analysis

1. Royalties from a company or supplier (The following conflicts were disclosed)

N/a

2. Speakers bureau/paid presentations for a company or supplier (The following conflicts were disclosed)

N/a

3A. Paid employee for a company or supplier (The following conflicts were disclosed)

N/a

3B. Paid consultant for a company or supplier (The following conflicts were disclosed)

N/a

3C. Unpaid consultants for a company or supplier (The following conflicts were disclosed)

N/a

4. Stock or stock options in a company or supplier (The following conflicts were disclosed)

N/a

5. Research support from a company or supplier as a Principal Investigator (The following conflicts were disclosed)

N/a

6. Other financial or material support from a company or supplier (The following conflicts were disclosed)

N/a

7. Royalties, financial or material support from publishers (The following conflicts were disclosed)

N/a

8. Medical/Orthopaedic publications editorial/governing board (The following conflicts were disclosed)

N/a

9. Board member/committee appointments for a society (The following conflicts were disclosed)

N/a

**Each author must sign AND print or type his/her name, date and submit a separate form**

In addition, one BLINDED Conflict of Interest form (no author names used) should be submitted per manuscript with all author disclosures.

Cameron Atkison 10/31/2024


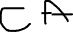


Author Name (Print or Type) Author Signature Date
